# Modified endoscopic hand-suturing without scope reinsertion for an ileocecal defect after endoscopic submucosal dissection

**DOI:** 10.1055/a-2523-7873

**Published:** 2025-03-25

**Authors:** Lizhou Dou, Shibo Song, Chen Zhang, Yumeng Liu, Ying Lv, Guiqi Wang

**Affiliations:** 1 Department of Endoscopy, National Cancer Center/National Clinical Research Center for Cancer/Cancer Hospital, Chinese Academy of Medical Sciences and Peking Union Medical College, Beijing, China; 2 26447 Endoscopy Center, Peking University First Hospital, Beijing, China; 3 Department of Endoscopy, National Cancer Center/National Clinical Research Center for Cancer/Cancer Hospital, Chinese Academy of Medical Sciences and Peking Union Medical College, Beijing, China; 4 Department of Endoscopy, National Cancer Center/National Clinical Research Center for Cancer/Cancer Hospital, Chinese Academy of Medical Sciences and Peking Union Medical College, Beijing, China; 5 Department of Endoscopy, National Cancer Center/National Clinical Research Center for Cancer/Cancer Hospital, Chinese Academy of Medical Sciences and Peking Union Medical College, Beijing, China; 6 Department of Endoscopy, National Cancer Center/National Clinical Research Center for Cancer/Cancer Hospital, Chinese Academy of Medical Sciences and Peking Union Medical College, Beijing, China; 7 Department of Endoscopy, National Cancer Center/National Clinical Research Center for Cancer/Cancer Hospital, Chinese Academy of Medical Sciences and Peking Union Medical College, Beijing, China

We present a modified endoscopic hand-suturing (EHS) technique that effectively and safely closed an ileocecal defect following endoscopic submucosal dissection (ESD) without requiring reinsertion of the endoscope. A 59-year-old woman underwent ESD for a laterally spreading tumor measuring approximately 3.5 × 3.0 cm in the ileocecum. The steps of the modified EHS procedure are detailed below ( Video 1Video 1 ).

An ileocecal defect created after endoscopic submucosal dissection was completely closed using a modified endoscopic hand-suturing technique in 59-year-old woman with a laterally spreading tumor in the ileocecum.Video 1Video 1

First, the 90° curvature of the V-Loc 180 needle (VLOCL0803; Covidien, Mansfield, Massachusetts, USA) was straightened to approximately 8° ( Fig. 1Fig. 1 ). This adjustment allowed the needle, along with the absorbable barbed suture, to fit within a polytetrafluoroethylene sheath tube with an inner diameter of 2 mm and an outer diameter of 2.5 mm ( Fig. 2Fig. 2 , Fig. 3Fig. 3 ). Additionally, the suture was shortened to facilitate the procedure.

**Fig. 1:**
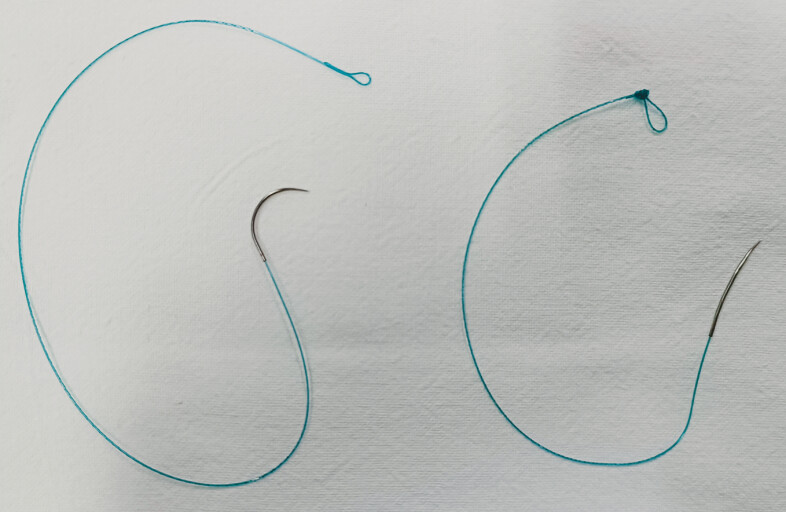
**Fig. 1** The original V-Loc 180 needle and the modified needle.

**Fig. 2:**
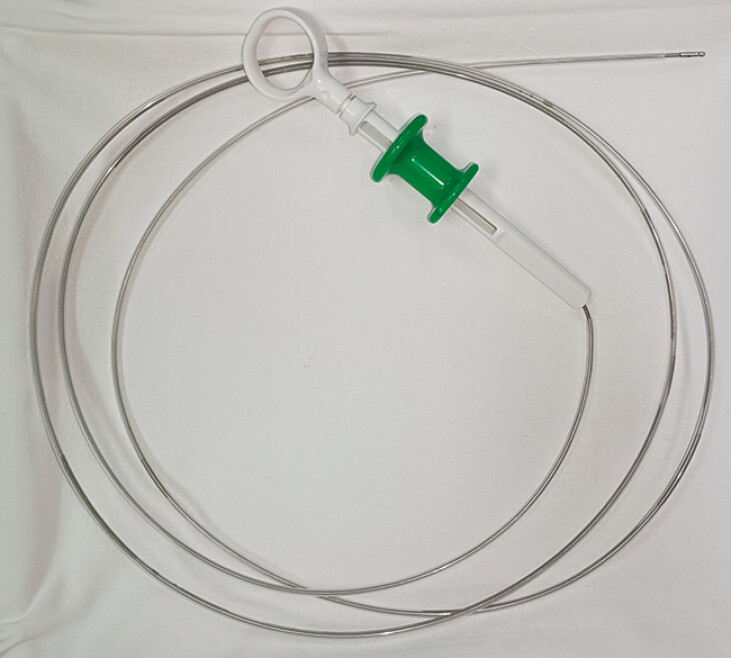
**Fig. 2** Sheath tube enclosing the biopsy forceps used in this case.

**Fig. 3:**
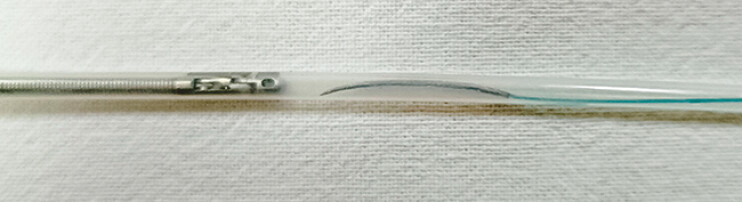
**Fig. 3** The modified needle with the absorbable barbed suture is placed at the front end of the sheath tube.

Next, the sheath was introduced into the ileocecum via the biopsy channel, and the needle was deployed into the intestinal lumen by advancing a 1.8 mm biopsy forceps within the sheath ( Fig. 4Fig. 4 ). A prototype needle holder, designed by our team, was then used to grasp the modified needle and perform linear continuous suturing to close the defect. Finally, the sheathed biopsy forceps were used to retract both the suture and needle back into the sheath for removal ( Fig. 5Fig. 5 ). The suturing process was completed in 20 min.

**Fig. 4:**
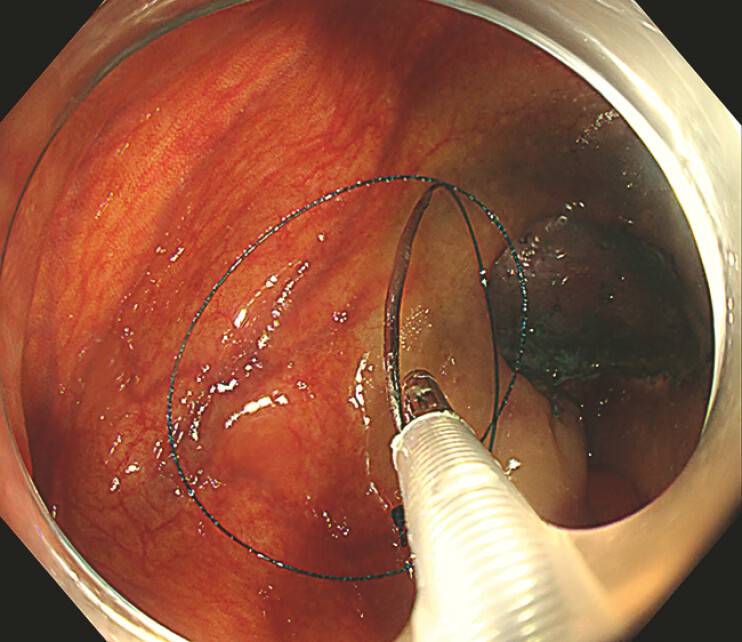
**Fig. 4** The needle is released into the intestinal lumen by advancing a 1.8 mm-diameter biopsy forceps within the sheath.

**Fig. 5:**
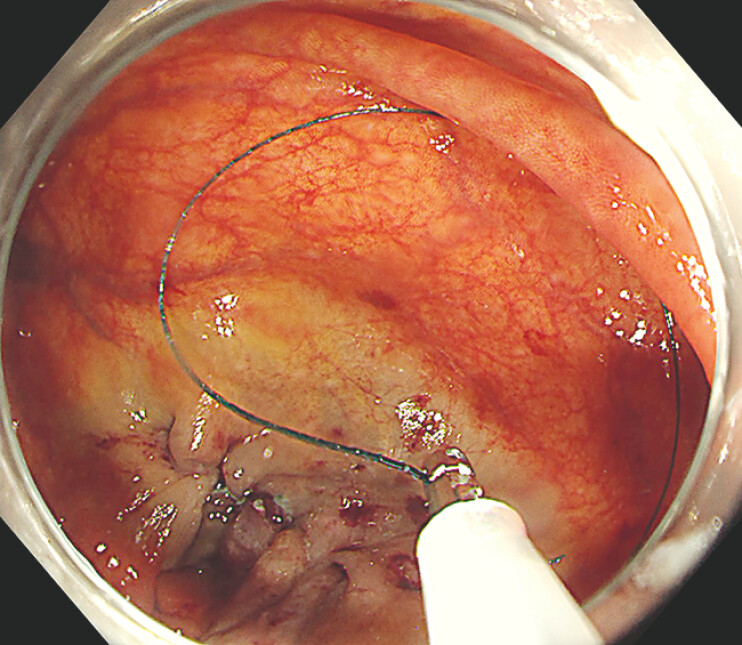
**Fig. 5** The remaining suture and needle are grasped and retrieved into the sheath by the sheathed biopsy forceps.

The patient was allowed to resume a liquid diet and was discharged on postoperative day 3 without any adverse events. Histopathological examination confirmed complete resection of a high-grade intraepithelial neoplasia. Follow-up endoscopy after 3 months demonstrated good healing of the defect.

The lack of a method for secure delivery of the needle makes EHS challenging to use in certain locations such as the proximal colon 11 22 33 . In this case, reducing the needle’s curvature and using a sheath system overcame this obstacle, eliminating the need for reinsertion of the endoscope. This case highlights the importance of thinking beyond conventional techniques when approaching endoscopic suturing.

Endoscopy_UCTN_Code_CPL_1AJ_2AJ

Citation Format

Endoscopy 2024; 56: E1022–E1023. DOI: 10.1055/a-2437-8238

## Notes

Endoscopy E-Videos https://eref.thieme.de/e-videos
*E-Videos* is an open access online section of the journal *Endoscopy* , reporting on interesting cases and new techniques in gastroenterological endoscopy. All papers include a high-quality video and are published with a Creative Commons CC-BY license. Endoscopy E-Videos qualify for HINARI discounts and waivers and eligibility is automatically checked during the submission process. We grant 100% waivers to articles whose corresponding authors are based in Group A countries and 50% waivers to those who are based in Group B countries as classified by Research4Life (see: https://www.research4life.org/access/eligibility/ ). This section has its own submission website at https://mc.manuscriptcentral.com/e-videos .
